# Beyond the label: ethical and clinical implications of off-label drug use in pediatric emergency care

**DOI:** 10.1186/s13052-025-02176-4

**Published:** 2025-12-13

**Authors:** Marco Colombo, Anna Plebani, Massimo Agosti

**Affiliations:** 1 Pediatric Emergency Department, Filippo del Ponte Hospital, ASST Sette Laghi, Varese, Italy; 2 Department of Medicine and Surgery, University of Insubria, Varese, Italy

**Keywords:** Off-label medications, Pediatric emergency medicine, Drug safety, Clinical practice, Informed consent, Adverse drug reactions, Drug regulation, Pediatric pharmacology, Evidence-based medicine, Healthcare policy

## Abstract

Off-label prescribing, the use of medications outside of approved indications, is a common practice in pediatric emergency medicine. This practice is driven by factors such as limited pediatric-specific clinical trial data, regulatory hurdles, and the urgent need to treat critically ill children. While off-label prescribing can be lifesaving, it raises significant ethical and legal concerns. This article explores the prevalence, challenges, and potential consequences of off-label drug use in pediatric emergency departments. A case study illustrates the complexities of off-label prescribing in a real-world clinical scenario. The discussion highlights the importance of balancing clinical needs with regulatory requirements and ethical considerations. Future research should focus on optimizing informed consent procedures, enhancing postmarketing surveillance, and developing evidence-based guidelines to ensure the safe and effective use of off-label medications in pediatric emergency care.

## Background

Off-label prescribing refers to the use of drugs outside of the approved indications, as specified in the Summary of Product Characteristics (SPC), regarding dosage (including frequency of administration), route of administration, age group, or disease/symptom [1].

Off-label drug use is common in pediatrics, particularly in NICUs and PICUs. A meta-analysis of 45 global studies revealed that 56% (95% CI, 0.46–0.66) of medications in these settings were prescribed off-label [2]. Pediatric emergency departments report that 25–30.5% of prescriptions are off-label [3]. Bronchodilators (e.g., salbutamol), nebulized adrenaline, and antiemetics (e.g., ondansetron) are frequently prescribed off-label [4, 5]. Salbutamol, for example, is often given off-label to children under 2 or at higher doses than recommended [6].

The extensive use of off-label medications is driven by challenges in conducting pediatric-specific studies, limited industry interest in pediatric indications, and postmarketing restrictions due to adverse events [7–9].

This review critically examines the controversies, challenges and established best practices of off-label drug use in pediatric emergency departments, contextualizing the discussion with an illustrative clinical case and delineating key priorities for future research.

## Case report

An 11-year-old male patient, 30 kg in body weight, with an unremarkable medical history, presented to the pediatric emergency department due to acute moderate asthma exacerbation, characterized by dyspnea, bronchospasm, ambient air oxygen saturation (SpO₂) of 90%, and a respiratory rate of 30 breaths per minute. The patient had been previously treated by his primary care pediatrician with three nebulizations of short-acting beta-agonists (SABA) at a dose of 4.5 mg each, which were administered every 20 min, two hours prior to hospital arrival, without clinical improvement.

Upon admission to the emergency department, 30 mg intravenous methylprednisolone was administered, along with three nebulizations of 2.5 mg salbutamol combined with 500 µg ipratropium bromide, adminisetred at 20-minute intervals. After four hours of observation, the patient was discharged with stable vital signs and was prescribed a home therapy regimen consisting of fluticasone 100 µg to be administered twice daily and SABA 2.5 mg three times daily for five days.

Three days later, the patient returned to the emergency department in good general condition, without signs of bronchospasm, seeking a second opinion regarding the SABA dosage, as his primary pediatrician had increased the dose from 2.5 mg to 4.5 mg per administration.

## Discussion

This case raises a significant clinical dilemma: in the context of a discrepancy between the therapy prescribed in the emergency department and the regimen subsequently modified by the primary care pediatrician, which therapeutic approach is most appropriate? This case underscores the need for reflection on shared criteria for drug dosing in emergency situations, especially in off-label use, prompting a discussion on the alignment of therapeutic responsibilities across care settings.

In Italy, salbutamol is the first-line medication for treating acute asthma exacerbations [10]. However, the dosages recommended by guidelines differ significantly from those provided on the drug label, which was last updated by the Italian Medicines Agency (AIFA) in October 2014.

The clinical case described above underscores the frequent off-label prescription of salbutamol in both primary care and pediatric emergency settings. The primary care pediatrician prescribed a dose exceeding the maximum indicated on the drug label for an 11-year-old patient (2.5 mg per dose), whereas the emergency pediatrician prescribed the drug off-label concerning the frequency of administration, despite the drug label specifying a maximum of four doses per day.

According to Italian law, physicians are safeguarded against legal liability when practicing in alignment with clinical guidelines and best practices [11]. However, they are also obligated to follow the summary of product characteristics (SmPC) which is an EU-wide harmonized document. Therefore this obligation applies uniformly across all member states, ensuring consistent prescribing standards throughout the European Union.

Since the indications within the SmPC often diverge from those recommended in clinical guidelines, physicians, assuming personal responsibility and obtaining informed consent from the patient, may prescribe medications off-label—even systematically and when therapeutic alternatives exist—provided that at least phase II clinical trials support the prescription and that it adheres to the scientific literature while ensuring safety and cost-effectiveness [12].

Furthermore, physicians are obligated to maintain a record of off-label prescriptions and to monitor and report adverse drug reactions (ADRs) [12].

The first Italian law addressing this matter dates back to 1996. At that time, the requirement for informed consent stemmed from the legislature’s aim to protect patients from the use of unauthorized therapies that might be potentially ineffective and carry a greater risk of adverse reactions. By contrast, although the FDA does not explicitly require informed consent for off-label prescribing, it is widely regarded as good medical practice and is frequently recommended [13]. Meanwhile, while the EU Court of Justice confirms that physicians may prescribe medicines off label, EU law forbids any promotion of such uses, leaving informed-consent requirements to be defined at the national level [14].

However, in emergency situations with severe impairment of vital functions, consent for treatment may be omitted, as established by patient rights conventions and the European Society of Emergency Medicine [15]. Notably, in pediatrics, consent must be provided by parents who, despite not being directly involved in the clinical emergency, may be in a psychological state that impedes their full comprehension of the information required for informed consent.

With respect to adverse drug reactions (ADRs), the literature reports that a 3.44–7.43-fold greater risk is associated with off-label drug use [16, 17]. Nonetheless, in the specific context of pediatric emergency departments, evidence is limited and somewhat contrary: the only study analyzing ADRs in this setting recorded a higher incidence of adverse reactions for on-label therapies than for off-label therapies (6.4 vs. 1.9 reactions per thousand prescriptions, respectively), with salbutamol being the most frequently implicated drug [5].

This apparent discrepancy may reflect several factors. First, off-label use in emergency settings predominantly involves well-characterized medications (e.g. salbutamol) employed for minor dose or age-related adjustments rather than novel indications, resulting in a more favorable safety profile. Second, the acute, short-term administration under intensive monitoring in the PED facilitates early detection and management only of short-term ADRs. Third, methodological differences—prospective vs. retrospective designs, variable definitions of off-label use, and thresholds for ADR reporting—can substantially influence incidence estimates. Finally, the severity of presenting conditions in the PED may justify higher risk-tolerance, leading clinicians to prioritize potentially life-saving interventions with known safety margins. Together, these elements underscore the need for context-specific pharmacovigilance strategies when interpreting off-label ADR data across different pediatric settings.

For these reasons, informed consent represents one of the most complex issues in daily pediatric emergency practice. Guidelines often recommend dosages and medications that differ from or are not covered by the official Summary of Product Characteristics (SmPC), creating potential ethical dilemmas in cases where parents refuse treatment.

Informed consent procedures should be adapted to the specific needs of pediatric emergency departments, where unlike other care settings, there is a high volume of patients and prescriptions but limited time available. This adaptation could include developing a standardized information sheet outlining general principles supplemented by a patient-specific information sheet for each off-label medication.

In many emergency care systems, local protocols explicitly allow for verbal or deferred consent in life-threatening situations. This approach reconciles the immediate need for urgent care with the absence or delay of traditional informed consent. A key solution lies in empowering physicians to obtain verbal consent when written consent would unduly delay critical treatment. This is particularly relevant in systems that accept verbal or retrospective consent, providing a legal and ethical framework for prioritizing patient well-being during emergencies. These protocols often stipulate that a more comprehensive, retrospective consent process should follow once the patient’s condition stabilizes, or that verbal consent be documented thoroughly, often witnessed, at the time it’s given. This ensures that while immediate life-saving interventions are not hindered, the principles of patient autonomy and transparent communication are upheld as soon as feasible.

Parents should be informed about the rationale behind off-label prescribing. It is essential to clarify that off-label medication is not necessarily inappropriate. While they may lack formal regulatory approval, such medications are prescribed on the basis of evidence-based guidelines and internal protocols to ensure both patient safety and therapeutic efficacy.

In China, the 2021 pediatric off-label guidelines [18] prompted an expert panel to issue an evidence-based framework—covering risk–benefit assessment, informed consent, expert-committee oversight, adverse-effect monitoring, and patient/family education—yet a 2024 survey found that clinicians rated informed consent documentation, educational materials, and an ADR-monitoring network as lower priorities, suggesting that in emergency pediatrics immediate life-saving interventions often supersede formal consent structures [19]. A parallel can be drawn in Europe: although the EU Pediatric Regulation and Italian laws (Di Bella Law 94/1998, L. 219/2017) legally ADR reporting for off-label prescriptions, they lack detailed, uniform processes or enforcement mechanisms, resulting in variable implementation and a tendency among healthcare professionals to favour urgent treatment over structured consent and monitoring pathways [14].

## Research agenda

Research priorities in pediatric emergency medicine should include the following:

### Evaluating the impact of informed consent

Investigate how parental refusal of consent for off-label medications influences clinicians’ therapeutic decisions and patient outcomes, with the hypothesis that denial of consent may be associated with suboptimal treatment efficacy and worsened clinical trajectories.

### Pharmacovigilance of off-label drug use

Enhance postmarketing surveillance frameworks to systematically detect, characterize, and quantify adverse events arising from off-label drug administration in pediatric emergency settings, thereby generating robust safety data to inform evidence-based prescribing guidelines.

These results could reopen the debate on the appropriateness of informed consent requirements for off-label use, particularly in pediatric emergency settings, leading scientific communities to call for alternative legislative frameworks that more effectively address the unique challenges of pediatric emergency care.

## Conclusions

In summary, the off-label use of medications in pediatric emergency medicine is a prevalent practice that, while critical therapeutic solutions are provided, raises significant ethical, legal, and clinical concerns. The existing deficit of standardized and current guidelines, compounded by the complexities of securing informed consent in emergent scenarios, necessitates a thorough reassessment by healthcare regulatory bodies and the scientific community. There is an urgent imperative to develop national evidence-based guidelines and pediatric formularies, enhance the distribution and promotion of AIFA notes among healthcare professionals, institute continuous professional development programs for healthcare practitioners and establish explicit protocols for informed consent to ensure the safe and judicious application of off-label medications. Concurrently, ddicated research grants and public–private partnerships—would reinforce clinical evidence generation for both on- and off-patent agents. Finally, the establishment of local pharmacovigilance committees and a formal pediatric ADR-monitoring network would ensure timely signal detection and feedback into practice guidelines. Together, these measures would create an integrated governance model that balances rapid therapeutic intervention with robust safety and quality assurance in pediatric care.

Future research endeavors should prioritize pharmacovigilance and rigorous risk analysis associated with these prescriptions, with the objective of optimizing the safety and efficacy of pediatric treatments. Only through an integrated and evidence-based paradigm can clinical practice be optimized, and the safeguarding of children within emergency settings be assured.

## Data Availability

Not applicable.

## References

[1] Frattarelli DA, Galinkin JL, Green TP, et al. Off-label use of drugs in children. Pediatrics. 2014;133(3):563–7. 10.1542/peds.2013-4060. 24567009 10.1542/peds.2013-4060

[2] Yuan X, Gao J, Yang L, Tan Y, Bajinka O. Off-label and unapproved pediatric drug utilization: A meta-analysis. Exp Ther Med. 2024;28(5):412. 10.3892/etm.2024.12701. 39268368 10.3892/etm.2024.12701 PMC11391174

[3] Balan S, Hassali MAA, Mak VSL. Two decades of off-label prescribing in children: a literature review. World J Pediatr. 2018;14(6):528–40. 10.1007/s12519-018-0186-y. 30218415 10.1007/s12519-018-0186-y

[4] CzarniakP,Bint L, Favié L, Parsons R, Hughes J, Sunderland B. Clinical setting influences off-label and unlicensed prescribing in a pediatric teaching hospital. PLoS ONE. 2015;10(3):e0120630. 10.1371/journal.pone.0120630. 10.1371/journal.pone.0120630 PMC4355417 25756896

[5] McD Taylor D, Joffe P, Taylor SE, et al. Off-label and unlicenced medicine administration to pediatric emergency department patients. Emerg Med Australas. 2015;27(5):440–6. 10.1111/1742-6723.12431. 26105103 10.1111/1742-6723.12431

[6] Venckute G, Zekaite-Vaisniene E, Oniunaite U, Jankauskaite L. Younger children with respiratory tract infections are more exposed to Off-Label treatments: an exploratory retrospective study in a pediatric emergency setting. Children. 2024;11(6):735. 10.3390/children11060735. 38929314 10.3390/children11060735 PMC11201448

[7] Valletta E, Gangemi M. Off-label drugs in pediatrics: from research to children and back again. 2022 Mar 22 [cited 2024 Oct 23]. Available from: https://www.farmacovigilanza.eu/node/15235 . Accessed 23 oct 2024.

[8] Agenzia Italiana del Farmaco (AIFA). Nuove importanti informazioni di sicurezza su Broncovaleas (salbutamolo). In Italian. Available from: https://www.aifa.gov.it/-/nota-informativa-importante-su-broncovaleas-salbutamolo-27-10-2014 . Accessed 23 oct 2024.

[9] Agenzia Italiana del Farmaco (AIFA). Modifica di indicazioni e popolazione autorizzata dei medicinali a base dell’associazione fissa (FDC) salbutamolo e ipratropio bromuro In Italian. 2023 Jul 5. Available from: https://www.aifa.gov.it/-/modifica-di-indicazioni-e-popolazione-autorizzata-dei-medicinali-a-base-dell-associazione-fissa-fdc . Accessed 23 oct 2024.

[10] Indinnimeo L, Chiappini E, Miraglia Del Giudice M, Bernardini R; Italian Panel for the Management of Acute Asthma Attack in Children. Guideline on management of the acute asthma attack in children by the Italian Society of Pediatrics. Ital J Pediatr. 2018;44(1):46. 10.1186/s13052-018-0481-1 10.1186/s13052-018-0481-1 PMC5889573 29625590

[11] Montanari Vergallo G, Zaami S. Guidelines and best practices: remarks on the Gelli-Bianco law. Clin Ter. 2018;169(2):e82–85. 10.7417/CT.2018.2059. 29595871 10.7417/T.2018.2059

[12] Agenzia Italiana del Farmaco (AIFA). Early access and off-label use. https://www.aifa.gov.it/en/accesso-precoce-uso-off-label

[13] Zain, Mithani. Virtual Mentor. 2012;14(7):576–81. 23351297 10.1001/virtualmentor.2012.14.7.oped1-1207

[14] Guidi B, Parziale A, Nocco L, et al. Regulating pediatric of-label uses of medicines in the EU and USA: challenges and potential solutions. Int J Clin Pharm. 2022;44:264–9. 10.1007/s11096-021-01303-5. 34495453 10.1007/s11096-021-01303-5

[15] EUSEM Ethics Committee. Recommendations on informed consent for European emergency departments for adults and children. https://eusem.org/images/Patient_Informed_Consent_in_the_ED-FINAL.pdf . Accessed 23 oct 2024.

[16] Horen B, Montastruc JL, Lapeyre-Mestre M. Adverse drug reactions and off-label drug use in pediatric outpatients. Br J Clin Pharmacol. 2002;54(6):665–70. 10.1046/j.1365-2125.2002.t01-3-01689.x. 12492616 10.1046/j.1365-2125.2002.t01-3-01689.x PMC1874497

[17] Pratico AD, Longo L, Mansueto S, et al. Off-Label use of drugs and adverse drug reactions in pediatric units: A Prospective, multicenter study. Curr Drug Saf. 2018;13(3):200–7. 10.2174/1574886313666180619120406. 29921210 10.2174/1574886313666180619120406

[18] Meng M, Liu E, Zhang B, et al. Guideline for the management of pediatric off-label use of drugs in China (2021). BMC Pediatr. 2022;22:442. 10.1186/s12887-022-03457-1. 35869466 10.1186/s12887-022-03457-1 PMC9307429

[19] Meng M, Hu J, Lei W, et al. The implementation of the guideline for the management of pediatric off-label use of drugs in china: a cross-sectional study. Transl Pediatr. 2024;13(8):1425–38. 10.21037/tp-24-198. 39263282 10.21037/tp-24-198 PMC11384445

